# Synergetic motor control paradigm for optimizing energy efficiency of multijoint reaching via tacit learning

**DOI:** 10.3389/fncom.2014.00021

**Published:** 2014-02-28

**Authors:** Mitsuhiro Hayashibe, Shingo Shimoda

**Affiliations:** ^1^INRIA DEMAR Project and LIRMM, University of MontpellierMontpellier, France; ^2^Brain Science Institute-Toyota Collaboration Center, RIKENNagoya, Japan

**Keywords:** feedback error learning, motor synergy, optimality, interaction torques, redundancy, Bernstein problem, tacit learning

## Abstract

A human motor system can improve its behavior toward optimal movement. The skeletal system has more degrees of freedom than the task dimensions, which incurs an ill-posed problem. The multijoint system involves complex interaction torques between joints. To produce optimal motion in terms of energy consumption, the so-called cost function based optimization has been commonly used in previous works.Even if it is a fact that an optimal motor pattern is employed phenomenologically, there is no evidence that shows the existence of a physiological process that is similar to such a mathematical optimization in our central nervous system.In this study, we aim to find a more primitive computational mechanism with a modular configuration to realize adaptability and optimality without prior knowledge of system dynamics.We propose a novel motor control paradigm based on tacit learning with task space feedback. The motor command accumulation during repetitive environmental interactions, play a major role in the learning process. It is applied to a vertical cyclic reaching which involves complex interaction torques.We evaluated whether the proposed paradigm can learn how to optimize solutions with a 3-joint, planar biomechanical model. The results demonstrate that the proposed method was valid for acquiring motor synergy and resulted in energy efficient solutions for different load conditions. The case in feedback control is largely affected by the interaction torques. In contrast, the trajectory is corrected over time with tacit learning toward optimal solutions.Energy efficient solutions were obtained by the emergence of motor synergy. During learning, the contribution from feedforward controller is augmented and the one from the feedback controller is significantly minimized down to 12% for no load at hand, 16% for a 0.5 kg load condition.The proposed paradigm could provide an optimization process in redundant system with dynamic-model-free and cost-function-free approach.

## 1. Introduction

A human motor system can continuously act to improve its behavioral performance toward optimal movement. Motor learning and control are executed seamlessly, adapting to environmental variations and newly-generated desired goals based on a person's intentions. In addition, when we move our limbs to execute a motor task, our body has more degrees of freedom (DOF) than the number of dimensions in its task space. Kinematic redundancy can contribute to better dexterity and versatility, but incurs an ill-posed problem of inverse kinematics from the task-description space to the human joint space. Such an ill-posed problem of DOF was originally formulated by Bernstein (1967) as the DOF problem. It is still an open problem on how motor controllers in the brain solve kinematic redundancy.

It is known that the cerebellum takes an important role in such motor learning by developing the internal model while comparing the actual outcome to the predicted outcome (Wolpert et al., 1998; Kawato, 1999). Ito (1972) first proposed that the cerebellum contains forward models of the limbs. This internal model theory has been well supported by behavioral studies in the field of motor control (Schweighofer et al., 1998) and by neurophysiological studies (Kawato, 1999). To establish such an internal model, feedback-error-learning (FEL) is well studied to provide computational adaptation paradigms, including prism adaptation, saccade adaptation and reaching (Kawato and Gomi, 1992a,b). There is extensive evidence that the learning system using feedback error relies on the cerebellum. FEL can provide an algorithm to establish the internal inverse dynamics model by minimizing the error against the desired joint angle trajectory. However, particularly for a redundant system, it does not provide a mechanism that can systematically improve performance toward optimal solutions such as minimizing total energy or torque changes (Uno et al., 1989). FEL has a computational adaptability, but for computational optimality in motor redundancy, FEL should be used together with the so-called cost function based optimization (Schweighofer et al., 1998; Todorov, 2004; Braun et al., 2009). In a typical approach to using FEL with a redundant system, the desired joint angle trajectory is prepared using optimization to solve the redundant problem, and FEL is then applied to establish the inverse dynamics model to map the joint angles and torques.

Several types of optimality model have been proposed. Such models are often defined as “minimum X,” where X can be jerk (Flash and Hogan, 1985), torque changes (Uno et al., 1989), motor command (Harris and Wolpert, 1998), and energy consumption (Alexander, 1997). In redundant manipulators, such cost function based optimal control was successfully applied in Todorov and Jordan (2002) and Guigon et al. (2007). In robotics, several methods were studied to deal with the redundancy (Nakamura, 1991; Nguyen-Tuong and Peters, 2011). They basically assume the use of a physical inverse dynamic model (Nakanishi et al., 2008) or approximation-based model (Peters and Schaal, 2008). The model-based cost function is commonly used for the optimization process. As for model-free approach, adaptive feedback control is already known in control society (Astrom, 1987; Marino and Tomei, 1993). Adaptive control is basically a mechanism of the parameter adjustment of the model or the gain adjustment of the controller using the trajectory error. However, adaptive control can not be applied to redundant systems without using cost function based optimization (Nakanishi and Schaal, 2004). In addition, dual task of the target task execution and the behavior optimization toward energy efficiency can not be performed in parallel.

The phenomenological optimal solutions appearing in human motion can be obtained using such a mathematical optimization approach. It is known that we employ muscle synergy (D'Avella et al., 2003; Alnajjar et al., 2013) for natural motion, which should have a relationship to optimal solutions in redundant space. Even if it is a known fact that an optimal motor pattern is employed phenomenologically, there is no evidence that shows the existence of a physiological process that may be similar to such a mathematical optimization in our brain or central nervous system (CNS). For instance, infants can modify their motion toward an optimal solution through repetitive interactions with the environment, but the appropriate cost function may initially not have been obtained. In addition, cost function based optimization is a process which involves a global image of the dynamic system over time rather than a simple feedback process of the states for an instant in time, which is a complex process to be embedded in the CNS as a modular configuration. Thus, we believe that there is an importance to find an alternative simpler computational paradigm which can induce equivalent optimization property. There are two types of redundancy in human motion. One is about muscle redundancy, and the other is about kinematic redundancy. In this study, we focus on the control solutions on kinematic redundancy, and we define a coordinated command pattern in the joint level as motor synergy.

In this study, we aim to find a more primitive computational mechanism to realize both adaptability and optimality in a redundant system with a dynamic-model-free and cost-function-free approach. A simple control architecture which can deal with optimality in a redundant system, can be a key organizational principle that CNS employs for achieving versatility and adaptability in motor control. FEL allows to establish the internal model, but does not provide an optimization process in a redundant system. Thus, the main contribution of this paper is to propose a new way of inducing optimization process without a prior knowledge of the system dynamics by using the task space error. Recently, a novel learning scheme named *Tacit Learning* was proposed (Shimoda and Kimura, 2010; Shimoda et al., 2013) as an unsupervised learning paradigm. Tacit learning is a biomimetic learning architecture where the primitive behaviors composed of reflex actions are tuned to the adaptive behavior. The experimental results demonstrated that the walking gait composed of primitive motions was well adapted to the environment in terms of walking efficiency (Shimoda et al., 2013). Here, we reformulated the paradigm as a supervised learning approach applied to simple cyclic reaching tasks using the feedback motor command error as a supervising signal.

This work is also oriented for reaching simulation of motor-impaired subjects and able-bodied subjects. The skeletal system has a complex series of linkages that produce coupled dynamics. For instance, when we quickly move our forearm by flexing the elbow joint, the flexion torques on the elbow joint accelerate our forearm. However, because of the forearm's inertia, this acceleration also produces torques on the shoulder. These interaction torques induce the undesired effect of accelerating the upper arm segment. The dynamics of multijoint limbs often cause such complex torques. However, an able-bodied subject can normally handle such interaction torques through motor learning and predict them without difficulty (Shadmehr and Wise, 2005; Braun et al., 2009). In contrast, the vertical reaching task was studied in patients with cerebellar lesions in Bastian et al. (1996). They concluded that cerebellar patients had specific deficits in their predictive compensation for the interaction torques. In control subjects, the elbow and shoulder joints rotated in a synergetic manner to compensate for the interaction torques (Gribble and Ostry, 1999). In a patient with cerebellar damage, it was difficult to control the endpoint of the arm in a synergetic way between multiple joints because of gravity and interaction torques. This implied that cerebellar damage affects the prediction of interaction torques that is normally based on the internal model established from motor learning. Thus, it is significant to enable the control simulation of motor performance for subjects who are successful in dealing with the interaction torques and those who can not manage. In this paper, the control simulation results with redundant actuators demonstrate that the proposed method can systematically produce motor synergies and energy efficiency while finding a way to compensate the interaction torques during multijoint reaching tasks.

## 2. Materials and methods

### 2.1. Vertical reaching and dynamics simulation

**Figure 1:**
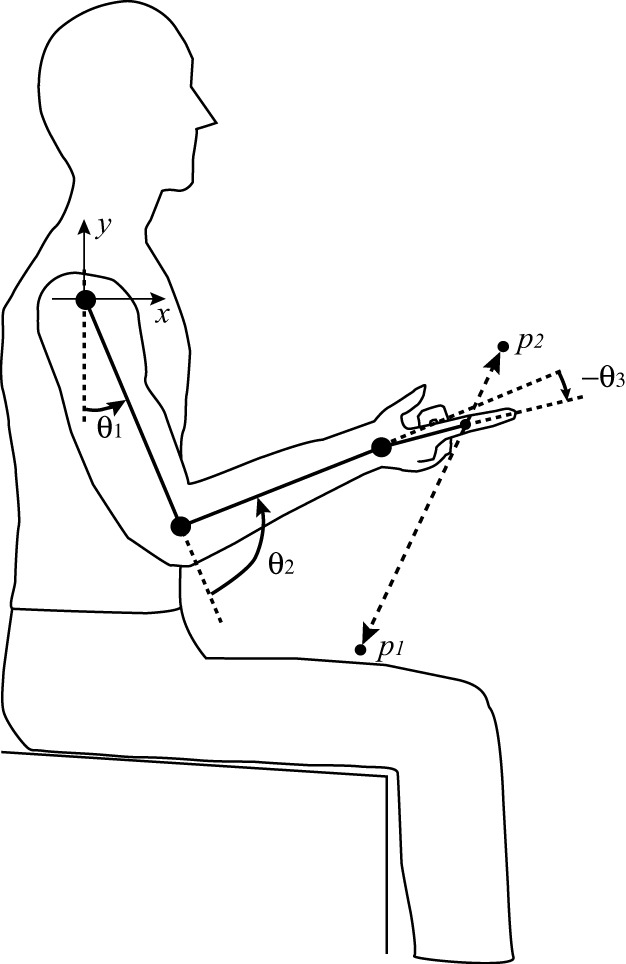
**Schematic representation of the vertical reaching task**.

In this study, we propose an optimal control paradigm in motor learning which has adaptability similar to FEL and optimality without using cost-function based optimization. We verified the performance of tacit learning in vertical reaching that involves complex interaction torques and the gravitational effect, as shown in Figure 1. This configuration was used in Bastian et al. (1996). We evaluated whether the proposed computational learning paradigm can learn how to compensate the interaction torques during multijoint reaching.

For simulating joint dynamics, we used MatODE (Caarls, 2010) which is a Matlab interface to the Open Dynamics Engine (Russell Smith, 2000). In a sagittal plane, 3DOF composed of the shoulder, elbow and wrist joint were assumed. The upper arm, forearm and hand segments were connected through each joint in the dynamics simulation environment. All the dynamics simulations were managed by external ODE package, in which the control module has access only to the control of each joint torque, and none to the manipulator dynamics model itself, in the learning process. It should be noted that the configuration used in this study is in the so-called Bernstein's DOF problem where we have actuation redundancy because the task is performed in 2D with a 3DOF manipulator.

### 2.2. Configuration of controller with tacit learning

In tacit learning, the command signal accumulations during repetitive interactions with the environment, play a main role in creating appropriate behavior. In biological controllers, signal accumulations can be considered as the typical learning method to create the adaptive behaviors, such as long-term depression (LTD) and long-term potentiation (LTP) in the cerebellum (Coesmans et al., 2004).

**Figure 2:**
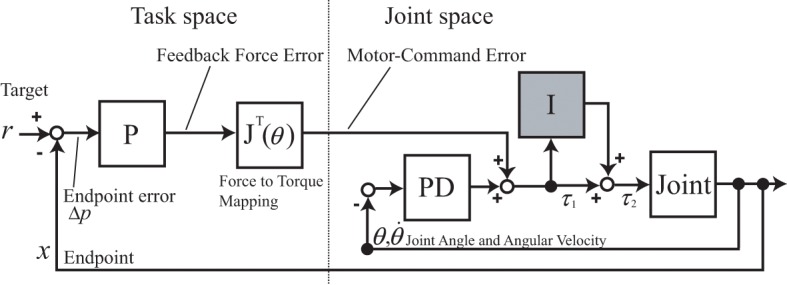
**A block diagram of motor learning for reaching motion using tacit learning**. P represents Proportional, D Derivative, and I the Integral controller, respectively. The box named joint represents the physical joint of the arm. The intention of the subject is expressed by a force vector in the task space, which represents the direction to the target and the distance as its intensity, using the proportional feedback error between the target and current endpoint. The feedback torque command error at each joint space is computed through the Jacobian of the arm by mapping the feedback force into the joint torque space. Local PD control represents the local reflex loop as a function of a muscle spindle. The torque command accumulation part in gray color corresponds to tacit learning.

Previously in tacit learning for biped walking, joints were divided into kinematically specified and unspecified groups (Shimoda et al., 2013). The unspecified joints were then controlled with tacit learning as an unsupervised learning paradigm. In this reaching task, only the desired position in task space was given as a target to follow, and all the joints were controlled with tacit learning as in Figure 2. The block diagram was formulated as a supervised learning paradigm using the feedback motor command error. Conceptually, it has an approach in common with FEL in how to use feedback errors as supervising signals. However, in FEL, optimization of some criteria is still necessary to achieve optimality. Thus, we aim to provide a primitive mechanism for realizing such optimality along with a FEL-like controller without using cost function. As in the mechanism of the cerebellum with regard to LTD and LTP, simple tacit learning with torque signal accumulation is employed to realize both adaptation and optimal control synchronously. This study is oriented for compensation of the interaction torques of unknown multijoint dynamics. We assume that only forward kinematics (FK) information is available. In contrast, in FEL, the given inverse kinematics computation is typically assumed to establish the internal inverse dynamics (ID) model.

Let us explain the details of the proposed learning paradigm shown in Figure 2 by listing separated steps:

1. The intention of the subject to follow the target is expressed by a force vector in the task space, which represents the direction to the target, and the distance as its intensity, using the proportional (P) feedback error between the target and current endpoint.
2. The feedback force error is mapped through the Jacobian of the arm into the joint torque space. It can be regarded as motor-command error that works as a supervising signal, as in FEL.
3. Local proportional derivative (PD) control mainly corresponds to a local reflex loop as a function of the muscle spindles. This part basically contributes to changing the joint angles smoothly.
4. Torque command accumulation part shown as gray box corresponds to tacit learning. This Integral (I) part serves as a unique learning process. This motor command accumulation part starts to learn how to compensate the interaction torques, and turns into a predictive torque patterns after the motor learning.

Specifically, the controllers for PD feedback and tacit learning can be expressed as follows.

PD feedback case:

(1) $$\tau_{1}=-J^{T}(\theta )k\Delta p-A\Delta \theta -B\dot{\theta }.$$

Tacit Learning case:

(2) $$\begin{array}{l} \tau_{2}=-J^{T}(\theta )k\Delta p-A\Delta \theta -B\dot{\theta }\;+\;C\int \;\tau_{1}dt.\\ \tau_{1},\tau_{2},\Delta \theta ,\dot{\theta }\in R^{m},\Delta p\in R^{n},J^{T}(\theta )\in R^{m\times n},A,B,C\in R^{m\times m} \end{array}$$

where, *m* is the number of the joints, *n* is the dimensional number of the task space, **τ** implies the control torque inputs of the joints, **θ** implies the angles of the joints, $\dot{\theta }$ implies the angular velocities of joints. ***J**^T^* (**θ**) is the transpose of the Jacobian of the arm, *k* is the gain of the task space propotional feedback, Δ***p*** is the endpoint error vector. This term corresponds to the neural substrate of force mapping functionality presumably due to corticospinal control (Bizzi et al., 1991).

***A*** and ***B*** are diagonal matrices which consist of the proportional and derivative gains of the PD controllers. ***C*** is a diagonal matrix which consists of the gains of the torque command integration regarding motor-command error and local feedback torque. The term ***A***Δ**θ** is optional, and it can be set if you specify the neutral position of the joint. In this simulation, this neutral position is specified only for the wrist joint, because the wrist tries to return to the neutral position when we relax.

As for local PD feedback, this part corresponds to a local reflex loop as a function of the muscle spindles (Shadmehr and Mussa-Ivaldi, 1994). When a muscle is stretched, primary sensory fibers of the muscle spindle respond to changes in muscle length and velocity. The reflexivity evoked activity in the alpha motoneurons is then transmitted via their efferent axons to the muscle, which generates force and thereby resists the stretch. This work is still at joint level representation, but the resisting feature against muscle length change and velocity change can be captured by the resisting feature in the joint angle and angular velocity changes as in local PD control.

Note that all joints are controlled independently, then this configuration can be regarded as a modular structure presumably implemented within cerebellar pathways. All dynamical parameters, such as segment inertia and mass, and the model itself, are completely blind to the controller. Differently from typical optimal solutions that is based on model-based cost functions, our approach is to produce such optimization process without using cost function, purely with repetitive interactions with the environment. It purely works only with the controller presented, that tends to find optimal solutions by the given dynamic environment. The difference between the PD feedback case and the tacit learning case is only the last term of the command signal accumulation in Equation (2).

### 2.3. Mechanism of tacit learning for reaching

As for the neurological explanation of the proposed control model, it has a common concept with FEL regarding the use of feedback error as a supervising signal. We can basically apply the same physiological roles as in FEL and the so-called internal model theory in the cerebellum (Kawato, 1999). The climbing fiber inputs to Purkinje cells carry error signals in the motor command coordinates, and their temporal waveforms can be well reproduced using the inverse dynamics model. The phase shift between feedback control and feedforward control during motor learning is well justified by obtaining the internal model in the cerebellum in previous papers (Kitazawa et al., 1998; Kawato, 1999). Feedforward movements are made without sensory feedback, which have predictive nature of the given dynamics. Feedback control, in contrast, involves modification of the current movement using information from sensory receptors and error detection. Optimal movement control likely reflects a combination of both feedback and feedforward processes (Desmurget and Grafton, 2000).

The difference in this work from a typical FEL configuration is first the point where the motor-command error is created by the mapping between the task space force and the joint space torque. In FEL, the optimized desired trajectory of position and velocity in joint space should be prepared in advance by optimizing some criteria specifically for the arm with redundant degrees of freedom (Schweighofer et al., 1998). Even if we use the Jacobian information, we do not perform inverse kinematic (IK) computation explicitly. The pseudo-inverse of Jacobian is not computed in this method differently from the typical methods in the robotics approach. Thus, the dimension reduction is not performed. The Jacobian itself can be obtained with the knowledge of the FK model. Thus, only FK information is assumed in this method, and the IK and ID models are unknown, then how to take the dynamics into account is being learned by the repetitive interactions with the environment. Thus, the controller design is different from a typical FEL configuration. In the proposed method, the optimality can also be addressed by tacit learning with command signal accumulations. Thus, along with the adaptivity originating from the FEL architecture, the optimal solution manageability can be a significant contribution of this study.

As for the explanation on how motor performance can be optimized over time, the motor command accumulation part serves as an energy feedback with task space directional information. Simply, in general error feedback control, when the error is feedback, the error can be minimized. Similarly, the integrated torque command contains an energy measure since it accumulates the past torque generation history during cyclic reaching task. Then this term works as directional energy feedback, thus naturally the energy can be minimized as it is in a feedback loop. Thus, tacit learning can induce energy minimization through the repetitive actions with the environment while minimizing the endpoint error toward a given target point in the task space.

### 2.4. Control simulation study of vertical reaching

The task of vertical reaching is to move the endpoint of the arm following the target which is moving between two points at a frequency *f* = 0.5 Hz. These two points in the coordinate system of Figure 1 and the moving target ***r***(*t*) are given as follows:

(3) $$\begin{array}{l} p_{1}={\left[\begin{array}{cc} 0.25 & -0.5 \end{array}\right]}^{T},\;p_{2}={\left[\begin{array}{cc} 0.35 & -0.1 \end{array}\right]}^{T}.\\ r(t)\;=(p_{1}-p_{2})\;\text{ sin}(2\pi ft)/2+(p_{1}+p_{2})/2.\; \end{array}$$

Initial joint angles are θ_1_ = 0°, θ_2_ = 90°, θ_3_ = 0°. As for the segment length, the inertia around the z axis and the mass of the upper arm, forearm and hand, they are obtained from the anthropometric table reported in De Leva (1996). They are set respectively as follows:

$$\begin{array}{l} l_{1}=0.282[\text{m}]\;l_{2}=0.269[\text{m}]\;l_{3}=0.086[\text{m}],\\ I_{1}=0.01275[{\text{kgm}}^{2}]\;I_{2}=0.006516[{\text{kgm}}^{2}]\;I_{3}=0.001305[{\text{kgm}}^{2}],\\ m_{1}\;=1.978[\text{kg}]\;m_{2}=1.183[\text{kg}]\;m_{3}=0.445[\text{kg}]. \end{array}$$

The control gains are set respectively as follows:

(4) $$\begin{array}{l} k=20.0,\;A=\left[\begin{array}{ccc} 0 & 0 & 0\\ 0 & 0 & 0\\ 0 & 0 & 0.05 \end{array}\right]\text{​},\;B=\left[\begin{array}{ccc} 0.01 & 0 & 0\\ 0 & 0.01 & 0\\ 0 & 0 & 0.01 \end{array}\right]\text{​},\\ C=\left[\begin{array}{ccc} 0.15 & 0 & 0\\ 0 & 0.15 & 0\\ 0 & 0 & 0.15 \end{array}\right] \end{array}$$

We investigate the motor learning with different loads at the endpoint. Two conditions, with no load or a 0.5 kg load attached to the hand, were evaluated. The energy consumption during each reaching cycle is compared between only PD control case and tacit learning controller in different load conditions. In this study, the 30% value of the above mass parameters both for arm segments and load, was used to achieve faster convergence of the learning within 60 s, to allow plotting of the whole range of the learning process in the limited space. Even in this condition, the allocated mass and inertial parameters create the effect of gravity and interaction torques. As long as these dynamical parameters are blind as set in this simulation, it does not influence the verification of the tacit learning performance except for the learning speed. The control gains are set the same for the two controllers and for the different load conditions.

### 2.5. Comparison to model-based optimization

The above proposed method is purely based on control with sensory feedback information and FK model without using a knowledge of manipulator dynamics. The standard solution for redundant system control is to use mathmatical optimization with dynamics model as described in introduction. Thus, we have also tried the model-based optimization to compare to the result of tacit learning. We first define the equation of motion of the manipulator with *m* revolve joints. The equation of motion of such manipulator can be described as follows (Nakamura, 1991):

(5) $$\tau =R(\theta )\overset{..}{\theta }\;+\;\frac{1}{2}\dot{R}(\theta )\dot{\theta \;}+\;S(\theta ,\dot{\theta })\dot{\theta \;}+\;g(\theta ),$$

where **θ**, $\dot{\theta }$, $\ddot{\theta }$ ∈ *R*^*m*^ implies the vectors of joint angle, angular velocities and acceleration, respectively. We assume that **θ** expresses the relative angles between neighboring links. ***R***(**θ**) ∈ *R*^*m* × *m*^ is the inertia matrix that is symmetric and positive definite. The eigenvalue of ***R***(**θ**) has the upper and lower bounds for any **θ** because all elements in ***R***(**θ**) are the constant or the trigonometric function of **θ**. ***S***(**θ**, $\dot{\theta }$) ∈ *R^m^* denotes centrifugal and Coriolis forces. ***g***(**θ**) ∈ *R^m^* is the gravitational component derived from the potential energy of the manipulator *U*(**θ**). All elements in ***g***(**θ**) are trigonometric functions of **θ**. The link length, link mass and inertia are set as indicated in the previous section.

The Matlab function fminunc was used to optimize the joint torque **τ** with the constraints of the endpoint at the desired trajectory. The inverse dynamics is available with closed-form explicit equations as in Equation (5). This allows the joint angle to be calculated from the torques and vice versa. Optimal control solutions were obtained by finding deterministic controls **τ**(*t*) = {**τ**_*i*_(*t*)} (*i* = 1…*m*) in [*t*_0_; *t_f_*] such that the cost function

(6) $$E=\sum_{i\;=\;1\ldots m}\int_{\left[t_{0};t_{f}\right]}\tau_{i}(t)dt$$

is minimum during the cyclic reaching task. Fifty discrete points per one reaching cycle of 2 s are used for the optimization process.

## 3. Results

### 3.1. Motor control with tacit learning

To evaluate the performance of the proposed tacit learning, we compare the control results for vertical reaching between **(A)** a PD feedback controller and **(B)** tacit learning with feedback controller. The task of vertical reaching is to move the endpoint of the arm following the target.

**Figure 3:**
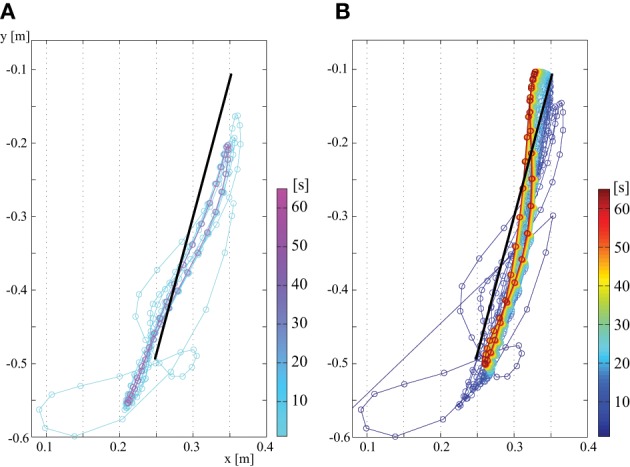
**Endpoint transition (no load at hand)**. **(A)** Only with feedback control and **(B)** with tacit learning in addition to feedback control.

Figure 3 shows a control result for vertical reaching. The first plot is the endpoint in the case with only PD feedback, and the second plot is with tacit learning in addition to feedback control. The time sequential transition is illustrated using a color map which changes depending on the progress of time. The color map correspondence to time can be seen in the color bar on the right side of figure. A cool color map is used for **(A)** PD feedback control, and a jet color map is used for **(B)** tacit learning. This colormap configuration is also used in other figures in this Result section.

**Figure 4:**
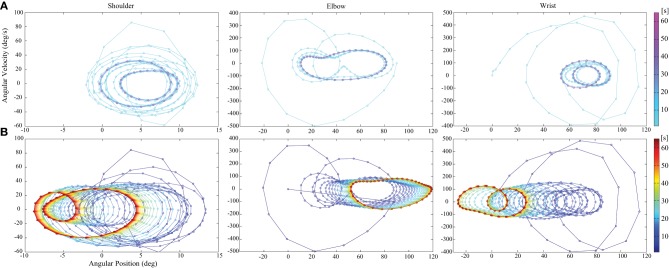
**Phase portrait of joint angle-angular velocity for the shoulder, elbow and wrist joints**. **(A)** With only feedback control and **(B)** with tacit learning in addition to feedback control. We see that each joint phase portrait in tacit learning is gradually shifted through the interactions with the environment from its original form that corresponds to the solution of only feedback case.

Figure 3 shows that PD control is largely affected by gravity and the interaction torques. In contrast, we see that the trajectory is corrected in time in the case of tacit learning, minimizing the effect of gravity and interaction torques. Figure 4 shows a phase portrait for the joint angle-angular velocity of the shoulder, elbow and wrist joints. We notice that each joint phase portrait in tacit learning is gradually shifted from its original form that is similar to the form in the PD controller. It seems that the joint space around the neutral position, which is θ_1_ = 0°, θ_2_ = 90°, θ_3_ = 0°., is being sought regardless of the effect of gravity and interaction torques.

### 3.2. Emergence of motor synergy via tacit learning

**Figure 5:**
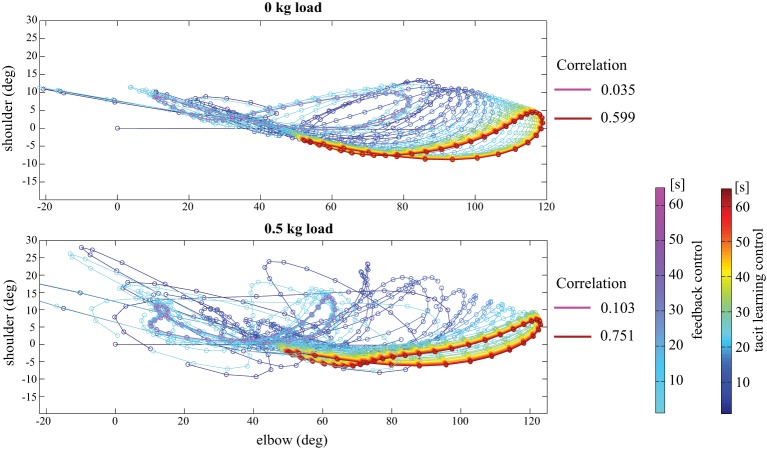
**Phase portrait between the shoulder and elbow joint angle in no load and 0.5 kg load conditions**. The line in the cool color map indicates the result with only feedback control, and the line in the jet color map is that for tacit learning. We see the phase in tacit learning converges into more aligned synergetic solutions compared with the case in only feedback control. As the metrics of the joint synergy, the coefficient of correlation between joint angles represents the synergetic joint usage in the tacit learning case.

Figure 5 indicates a phase portrait of the shoulder-elbow joint angle in no load and 0.5 kg load conditions. We can find that the phase in tacit learning converges into more aligned synergetic solutions compared with the case with only feedback control. The phase form in PD control changed with the addition of a 0.5 kg load. It represents that the controller is significantly influenced by gravity and the interaction torques.

As a result, it shows more unrelated and non-synergetic solutions between the shoulder and the elbow for PD control. In contrast, the phase form is similar for different load conditions in tacit learning. The shoulder and elbow joints are used in a synergetic way even with the load. As the metrics of the joint synergy, the coefficient of correlation between joint angles is calculated as in Figure 5. It showed low value in both PD control cases, in contrast, it showed high value after the learning process in tacit learning case. This implies that tacit learning allowed it to learn how to manage the interaction torques and to find synergetic combinations between neighboring joints to achieve efficiency in multijoint coordination. It is interesting to see such a synergetic solution is gradually found with the dynamics-model-free and cost-function-free approach. “synergistic solutions” can be considered as equivalent to “reduced space coordination.” The aligned solutions toward reduced dimension was appeared in different load conditions with tacit learning. It implies that the aligned solutions is also robust to the dynamic condition changes, since the phase form is not necessary to be largely modified.

**Figure 6:**
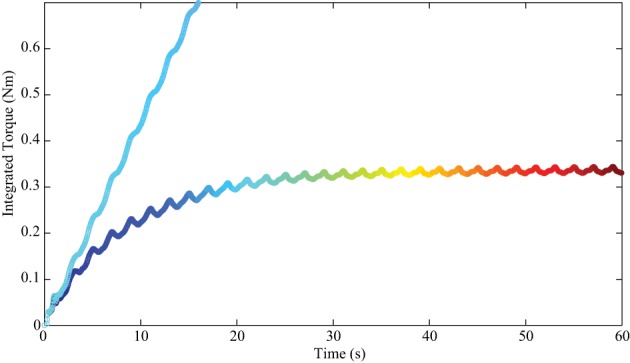
**The transition of integrated torque in the wrist joint (no load at hand)**. The line in the cool color map indicates the result with only feedback control. The integrated torque term was computed for comparison. The line in the jet color map is that for tacit learning. We see that the integrated torque pattern is converging into certain form that can be regarded as the predictive part which compensates gravity and interaction torques of the multijoint dynamics.

The integrated torque term of the wrist joint is depicted in Figure 6. The plot in the cool color map represents the case with PD feedback. In this mode, there is no integrated torque term in the controller, but this term was computed for comparison. The plot in the jet color map is the integrated torque term in tacit learning. We can find that the torque pattern of this term is converging into a certain form as in Figure 6. This torque pattern can be regarded as the part that compensates for gravity and the interaction torques of the dynamic system. In this sense, this torque integration term can be considered a feedforward (FF) controller which anticipates the environmental interactions during the reaching task. Motor learning is a process that develops a feedforward controller and minimizes the contributions from the feedback controller. During learning, the contribution from FF increased and the torque from FF converged into a certain pattern. Thus, tacit learning naturally matches this neurological learning process.

### 3.3. Energy efficiency and tracking error minimization

**Figure 7:**
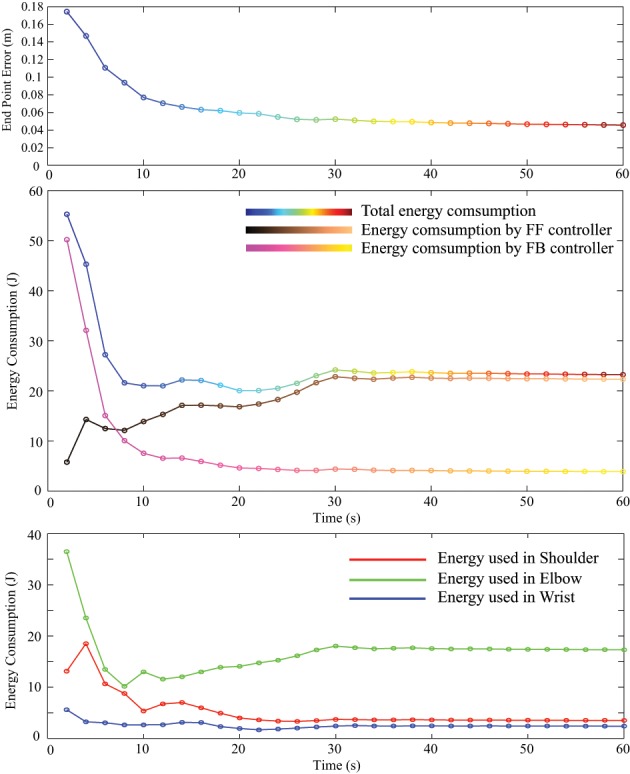
**The transition of endpoint error (top) and energy consumption in one cycle of reaching with a 0.5 kg load with tacit learning (middle)**. Not only improving the target tracking accuracy, but tacit learning solutions result in efficient total energy consumption. In addition, it was possible to observe the contribution ratio was switched between FB and FF controllers. Initially FB was mainly used, and with learning progress, the energy consumption with FB is significantly minimized. In addition, we notice that the energy used in the elbow increased while that in the shoulder decreased (**bottom**).

**Figure 8:**
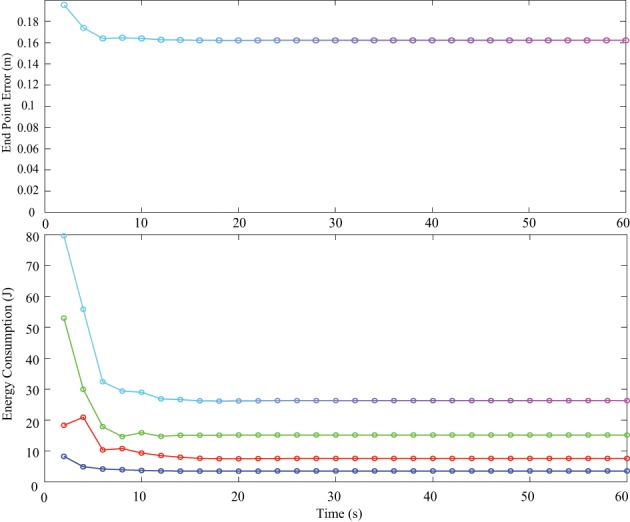
**The transition of endpoint error (top) and energy consumption in one cycle of reaching with a 0.5 kg load with feedback control case (bottom)**. Initial transition phase is purely due to the stored energy in the mass-spring-damping property by PD control, since the arm starts to move from the stopped condition toward the moving target.

Energy consumption in one cycle of reaching was measured and compared between PD control and tacit learning in different load conditions. The vertical reaching in this study was the motion between two vertically located points at a frequency of 0.5 Hz. Therefore, the energy consumption during every 2 s period was calculated by summing each joint energy consumption 2πτ$\dot{\theta }$. The transition of energy consumption in tacit learning with a 0.5 kg load is illustrated in Figure 7 (middle). The corresponding endpoint error is also plotted with the calculation of the root-mean-square (RMS) error between the target point and current endpoint during one cycle as in Figure 7 (top). The energy used in each joint is depicted by the red line for the shoulder, green for the elbow, and blue for the wrist joint as in Figure 7 (bottom). In Equation (2), the torque component of PD feedback can be regarded as a feedback (FB) controller, and the integration term can be regarded as a FF controller. The energy consumption by each torque component was also computed, as shown in Figure 7 (middle). For comparison, the transition of endpoint error and the energy consumption with a 0.5 kg load with feedback control case is shown in Figure 8. Initial transition phase is purely due to the stored energy in the mass-spring-damping property by PD control, since the arm starts to move from the stopped condition toward the moving target. Except this period, there is no adaptation process both in end point error as well as energy consumption. From the graph in Figure 7, the endpoint error is minimized asymptotically during motor learning. The energy consumption is also minimized globally, while the contribution from the FF controller is augmented in the course of motor learning and the contribution from the FB controller is minimized. In addition, we notice that the energy used in the elbow increased, while that in the shoulder decreased.

The endpoint error and energy consumption transition versus time is summarized in Table 1 for all conditions. We can notice that the energy consumption in tacit learning is minimized during motor learning, while the energy transition remains constant in the case of feedback control because there is no adaptive functionality. As for endpoint error, it was also minimized to improve target tracking accuracy in tacit learning, while it remained constant in FB control. The energy consumption ratio by the FF controller was augmented, while that of the FB controller decreased. The figures in parentheses in Table 1 indicate the cycle-to-cycle variability to evaluate the convergence of tacit learning. We can confirm that the error, the energy and the contributions of FF and FB are all converged in the course of the optimization process in tacit learning.

**Table 1:** **Endpoint RMS error (m) and energy consumption (J) in one cycle of reaching**.

**Time (s)**	**PD**		**Tacit**				**High-gain PD**	
	**Error**	**Energy**	**Error**	**Energy**	**FF**	**FB**	**Error**	**Energy**
**0 kg**								
4	0.108	16.44	0.079 (0.0596)	15.71 (15.78)	5.42 (2.82)	10.33 (18.84)	0.030	19.51
10	0.107	15.12	0.043 (0.0078)	15.11 (0.14)	10.35 (1.33)	4.95 (1.15)	0.030	17.93
14	0.107	14.99	0.033 (0.0041)	15.68 (0.18)	12.60 (0.95)	3.31 (0.75)	0.030	17.92
20	0.107	15.07	0.027 (0.0014)	15.61 (0.06)	13.98 (0.30)	2.15 (0.26)	0.030	17.92
40	0.107	15.06	0.023 (0.0002)	15.25 (0.03)	14.61 (0.01)	1.87 (0.01)	0.030	17.92
70	0.107	15.06	0.022 (0.0)	15.05 (0.01)	14.41 (0.01)	1.82 (0.0)	0.030	17.92
**0.5 kg**								
4	0.174	55.80	0.147 (0.0411)	45.43 (9.99)	14.33 (8.56)	32.17 (18.17)	0.048	36.19
10	0.164	29.01	0.077 (0.0169)	21.08 (0.57)	13.92 (1.77)	7.56 (2.55)	0.047	31.20
14	0.163	26.62	0.066 (0.0042)	22.25 (1.18)	17.14 (1.80)	6.61 (0.03)	0.047	31.20
20	0.162	26.20	0.059 (0.0027)	20.10 (1.10)	16.87 (0.19)	4.63 (0.56)	0.047	31.20
40	0.162	26.32	0.049 (0.0008)	23.71 (0.16)	22.61 (0.17)	4.11 (0.04)	0.047	31.20
70	0.162	26.32	0.045 (0.0001)	23.20 (0.02)	22.30 (0.01)	3.85 (0.01)	0.047	31.20

The figures in parentheses indicate the cycle-to-cycle variability to evaluate the convergence of tacit learning.

In high-gain PD, the four times larger gain *k* of the task space propotional feedback is used. Even though the end point error is similar scale to the one in tacit learning, the energy consumption becomes larger. In contrast, the end point error is being minimized as well as the energy consumption in tacit learning. Between PD and Tacit, the same gain *k* of the task space propotional feedback is used.

We should note that more energy was naturally necessary when we try to follow the moving target more precisely. The absolute energy consumption was not significantly different between the only feedback and the tacit learning cases. However, we should remember that the tracking error was largely different in both cases.

To realize accurate target tracking performance with only PD control, much greater energy will be required by increasing feedback gain because the dynamic effect should be canceled precisely, while having the conflicts between joints. With a load of 0.5 kg in tacit learning, both accuracy and total energy clearly improved with synergetic motor control of the shoulder and elbow joints, as a result of the optimality in tacit learning. In high-gain PD case, the four times larger gain *k* of the task space propotional feedback is used. Even though the end point error is similar scale to the one in tacit learning, the energy consumption becomes larger. Between PD and Tacit, the same gain *k* of the task space propotional feedback is used.

**Figure 9:**
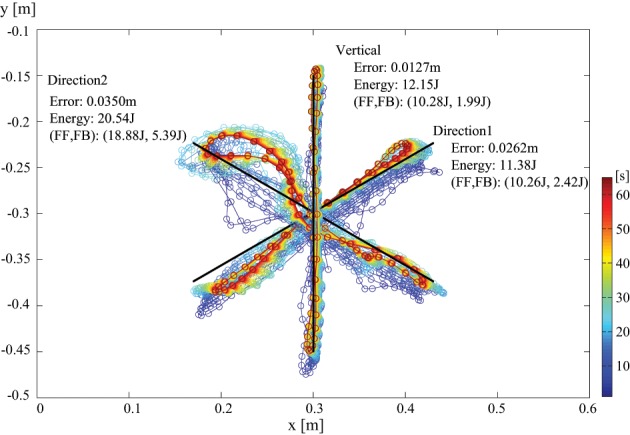
**Endpoint transition (no load at hand) for multidirectional reaching with tacit learning**. The target moving line was tilted with every 60°. For each different target direction, each learning is started from center position with no prior knowledge. The resultant error of the end point and the energy consumption is indicated as well as the contribution information of FF and FB controllers.

In addition, to show the performance for other tasks in different directions, a result for multidirectional reaching is shown as in Figure 9. The target moving line was tilted with every 60° for 3 directions. For each different target direction, the learning is started from center position with no prior knowledge. Here, the same control gains were employed for all the cases. Thus, we can find that the dynamics effect appears differently for different directions. For instance, the error and the energy consumption was different for each direction. Especially, direction 2 required larger energy than other directions. If we remind that the shoulder position is at origin of the coordinate, the inertial momentum around the shoulder and the swing-up momentum around the elbow can not be used effectively for direction 2, then we imagine that it was resulted in higher energy requirement.

### 3.4. Result with model-based optimization

We analyzed the system also with model-based optimization by using a knowledge of the dynamics model. The endpoint error and energy consumption with the solution produced by model-based optimization are summarized in Table 2. We can notice that the energy consumption is similar to the one converged in tacit learning. It implies that the solution in tacit learning is close to the optimal solution by the so-called optimization approach. If we remind that in tacit learning, no dynamics knowledge is used differently from the case in so-called optimization, the result supports the advantage of the proposed method.

**Table 2:** **Endpoint error and energy consumption in the case of model-based optimization**.

**kg**	**Error (m)**	**Energy (J)**
0	0.019	16.51
0.5	0.019	22.65

## 4. Conclusions and discussion

In this paper, we proposed a novel computational control paradigm in motor learning for a reaching task, especially vertical reaching which involves the management of interaction torques and gravitational effects. From the control results, we claim that the proposed method is valid for acquiring motor synergy in the system with actuation redundancy. We highlighted that tacit learning provides computational adaptability and optimality with dynamic-model-free and cost-function-free approach, in contrast to previous studies. Energy efficient solutions were obtained by the emergence of motor synergy in the redundant actuation space. Not only were the target tracking accuracy and energy efficiency improved, but the learning behavior was supported by a finding that the shift of contributions between the FB and FF controllers is observed, as shown in Figure 7 (middle). Phenomenologically, this shift fits well with the findings reported in the internal model theory (Kawato, 1999).

Finally, the FF torque pattern converged to a specific temporal pattern in order to manage the given dynamics, as shown in Figure 6. Such effect of command signal accumulation may be regarded as phenomenological LTP and LTD, provided by the tacit learning. The above explanation is a qualitative interpretation. As for a theoretical explanation of the learning process, motor command accumulation part served as an energy feedback with task space directional information. As same as the case where the error can be minimized in error feedback loop, energy could be minimized when the integrated torque command was in the feedback loop through the repetitive actions with the environment.

In this work, FEL was taken as an example to be contrasted with the proposed method, but the result of this work actually doesn't conflict with FEL at all. On the one hand, the proposed method can be regarded as a special form of FEL. On the other, a neural network FEL architecture (Kawato et al., 1987) is still useful to memorize the optimal control solutions in the obtained behavior together with sensory feedback signals for managing discrete movements. Thus, the proposed method could coexist alongside the conventional neural network FEL by adding a new optimality feature of the proposed method in a complementary role.

In addition, the simulation results showed good correspondence to the experimental results reported in Bastian et al. (1996). In their experiment, they found that the inability to produce accommodating joint torques for the dynamic interaction torques appeared to be an important cause of kinematic deficiencies shown by subjects with cerebellar abnormalities. Thus, the reaching by them showed lack of coordination of the shoulder and elbow joints, and a curved endpoint trajectory according to Bastian et al. (1996). The characteristics of reaching in subjects with cerebellar abnormality are equivalent to the results of **(A)** with only FB control in this study. In the case of PD feedback, we can confirm the failure to compensate for gravity and the interaction torques in Figure 3, and the curved non-synergistic joint use in Figure 5, which showed the conflicts between the joints. The level of the interference was higher in a 0.5 kg load condition because the interaction torque levels got higher.

In contrast, the experimental reaching of an able-bodied subject showed the correspondence to the result **(B)** with tacit learning. In Bastian et al. (1996), they suggest that a major role of the cerebellum is in generating joint torques with prediction of the interaction torques being generated by other moving joints and compensating for them. It implies that the proposed computational learning paradigm well represents the learning principles actually taking place in the cerebellum. The failure to manage interaction torques leads to the situation where one joint motion affects the motion of another. Thus, the solution to managing these environmental forces should be achieved by finding synergetic use of neighboring joints. When the conflicting torque could be minimized, it can naturally result in energy effective motion. It can be a reason why we employ motor synergy that can reduce the interaction conflicts in a multijoint system. In the results, the energy used in the elbow increased, while that in the shoulder decreased. This is one of the results from the strategy where an energy effective solution is being learned because the joint angle acceleration in the shoulder involves all the arm segments from the upper arm to the hand, while the joint angle acceleration in the elbow involves only the forearm and the hand, which are half the total mass in the arm dynamics system. Tacit learning found it only by repetitive interactions with the environment without using a dynamic model and cost function. This process is similar to human motor control principles, where even an infant can improve his motor control ability by repetitions without thinking about it. Increasing the contribution of the FF controller that corresponds to the so-called internal model development also matches well the nature of computational motor learning in a human being (Kawato, 1999). In Table 1, the contribution ratio was switched between FB and FF, initially FB was fully used, and with learning progress, the energy consumption with FB is significantly minimized down to 12% for no load, 16% for a 0.5 kg load condition. Instead, the contribution of FF is increased from initial 0% to 95% for no load, 96% for a 0.5 kg load condition. Since there is still remained conflicts between FB and FF solutions, the sum of both goes over 100%.

The results demonstrated in this paper also concern Bernstein's DOF problem. The Bernstein problem is how the CNS can find an optimal solution with actuation redundancy. The use of motor synergy was pointed out by Bernstein, but a fundamental motor control principal that can generate motor synergy has not yet been reported in neuroscience. In this study, it is a simple situation of actuation redundancy, but the proposed tacit learning first managed to generate motor synergy by a simple computational principle, which is more likely to be embedded as a modular configuration in the CNS, rather than the so-called cost function based mathematical optimization approach. The obtained solution in tacit learning also showed the similar energy consumption to the case with such model-based optimization.

In this study, we have not conducted the tuning of control gains, since we preferred to propose a new type of computational paradigm which can manage redundant system optimization process. However, in the given dynamic conditions and the given control gains, the tacit learning showed that the contribution of the FF controller is augmented in the course of motor learning and the contribution from the FB controller is minimized for all the directions and different loads. Further, more optimal solutions may be obtained in different control gain conditions. However, the detailed analysis on the dynamic stability of the system, would be required for the generalization of the control gain tuning for future work. Simple cyclic reaching task was used in this study to show the optimization process in redundant system. Toward complex task management, additional work would be necessary to establish the internal model from the optimized torque solutions obtained from the proposed method.

A recent study from the group of G. Courtine (Van Den Brand et al., 2012) reported that smart circuits embedded in the brain stem and spinal cord, may elaborate the detailed motor command toward optimum motor states, based on the supraspinal signal, current limb position, and constraints. As the proposed controller has a simple modular paradigm with a distributed structure that can be embedded into the individual controllers for multijoint coordination to achieve adaptivity and optimality for the total system, the proposed computational principle may also help to represent spinal adaptivity toward optimal solutions.

### Conflict of interest statement

The authors declare that the research was conducted in the absence of any commercial or financial relationships that could be construed as a potential conflict of interest.
